# The Smashed-Up Shoulder: A Case of Complex Ipsilateral Scapula, Clavicle and Proximal Humerus Fractures With Complete Brachial Plexus Injury

**DOI:** 10.7759/cureus.19918

**Published:** 2021-11-26

**Authors:** Ahmad Arieff Atan, Zamri Ab Rahman, Khairul Rizal Zayzan, Norhaslinda Bahaudin, Abdul Rauf Ahmad

**Affiliations:** 1 Orthopaedics, Hospital Tuanku Ja'afar Seremban, Negeri Sembilan, MYS; 2 Orthopaedics, Serdang Hospital, Selangor, MYS

**Keywords:** brachial plexus injury, floating shoulder, ipsilateral, humerus, clavicle, scapula

## Abstract

Simultaneous ipsilateral fractures involving all the bones around the shoulder girdle, namely, the scapula, clavicle and humerus, are rare. We describe an interesting case of a 31-year-old patient who presented after a motor vehicle accident with excruciating pain over his left shoulder and a flail left upper limb. Radiographs and computed tomography (CT) scan revealed the presence of comminuted left scapula, clavicle and proximal humerus fractures. He was also diagnosed with a complete brachial plexus injury of the left shoulder. The patient underwent a tedious surgery involving screw fixation and plating of the scapula, clavicle and proximal humerus. Despite achieving stable fixations of the shoulder and radiographic union of all the fractures, he did not recover from the complete brachial plexus injury 14 months after the trauma. The presence of ipsilateral clavicle, scapula and humerus fracture suggests involvement in high-energy trauma, and therefore, associated injuries especially neurovascular compromise should not be missed. Despite its rarity, management of this complex injury should always be individualised to ensure optimal functional outcomes are achieved.

## Introduction

High-velocity trauma has always been associated with a high morbidity and mortality rate. Shoulder girdle fractures are usually the result of such trauma, with the term “floating shoulder” coined to describe any dual injury to the superior shoulder suspensory complex (SSSC), which causes loss in the stability of the shoulder [1]. However, triple injury involving all bones around the shoulder girdle, namely, the scapula, clavicle and humerus, is believed to be extremely rare.

We describe an interesting case of a patient who survived after a high-impact motor vehicle accident and sustained complex ipsilateral fractures of the scapula, clavicle and proximal humerus with a complete brachial plexus injury on the affected limb. This case was previously accepted and presented as a virtual oral case presentation during the 50th Malaysian Orthopaedic Association Annual Scientific Meeting 2021 on June 22-25, 2021, and the 21st Asia Pacific Orthopaedic Association Congress Meeting 2021 on July 29-31, 2021.

## Case presentation

A 31-year-old male presented after a head-on collision motor vehicle accident, with a full Glasgow Coma Scale (GCS) score (15/15) and stable haemodynamically. He complained of excruciating pain over his left shoulder, with the inability to move his entire left upper limb.

Physical examination revealed multiple bruises and abrasions over the anterior and posterior aspects of the left shoulder. He was unable to move the whole left upper limb, with muscle power grading of 0 for muscle group innervated by C5 through T1 nerve roots. There was also sensory anaesthesia for the dermatomal distributions of C5-T1. No Horner’s syndrome was present to suggest preganglionic injury of the brachial plexus. The brachial, ulnar and radial pulses were palpable at good volumes and had triphasic waveform on Doppler ultrasound. No injuries to the chest, abdomen or other limbs were observed.

**Figure 1:**
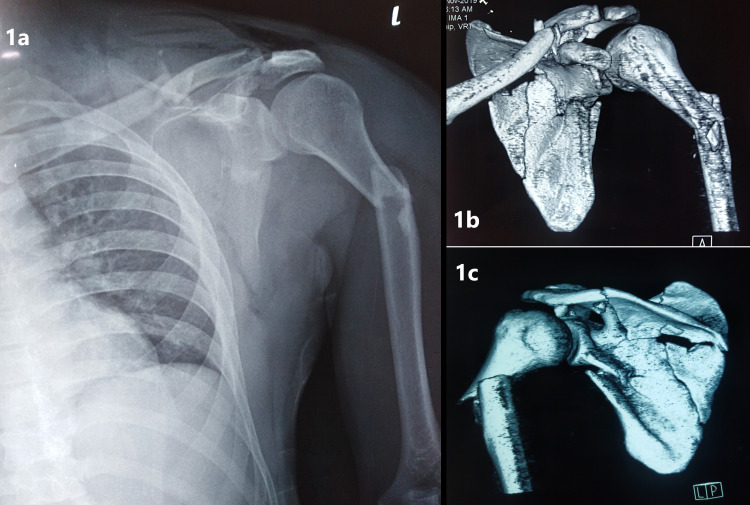
Anteroposterior (AP) radiograph and 3D-reconstructed CT scan images of the left shoulder. (a) The preoperative radiograph showing the anteroposterior (AP) view of the left shoulder clearly enabled the establishment of the diagnosis of the ipsilateral lateral third clavicle, comminuted scapula and proximal third humerus fractures. (b,c) Detailed assessment using CT scan showed both the anterior and posterior surfaces of the left shoulder in the coronal plane. The extension of the fractures can be clearly seen, especially for the scapula, which outlined the highly comminuted scapular fractures involving the scapular body, segmental fracture of its medial border, infraglenoid fracture of its lateral border and fracture of the scapular spine at the base of the acromion. This is indispensable for preoperative planning and preparation.

Subsequent radiographs showed that there were ipsilateral left scapula, clavicle and proximal humerus fractures. Further assessment using computed tomography (CT) scan revealed the highly comminuted scapular fracture involving its body, lateral and medial borders, and the scapular spine. There were also comminuted fractures of the lateral third clavicular shaft and extra-articular proximal humerus. The left acromioclavicular joint was surprisingly intact (Figure 1).

The decision to stabilise the shoulder girdle by fixation of all fractures was then made after considering the complete brachial plexus injury. The soft tissue contusion was allowed to subside first, and the abrasions were healed at the time of surgery, around 10 days after the trauma.

In the operating theatre, the patient was placed under general anaesthesia and was positioned in floppy lateral decubitus. This allowed access to both the anterior and the posterior aspect of the shoulder girdle. The whole shoulder girdle until the fingers were cleaned and prepped freely. The bony landmarks of the proximal humerus, clavicle and scapula were marked. The most distal bone, which is the humerus, was chosen to be operated upon first. A simple deltopectoral approach was adopted, and the proximal humerus was fixed using a proximal humerus 3.5 mm locking plate. The left clavicle was operated upon next, for which a direct clavicular approach was utilised, taking special consideration into preserving the supraclavicular nerves. The clavicle was then fixed using a 3.5 mm anatomical clavicular locking plate.

**Figure 2:**
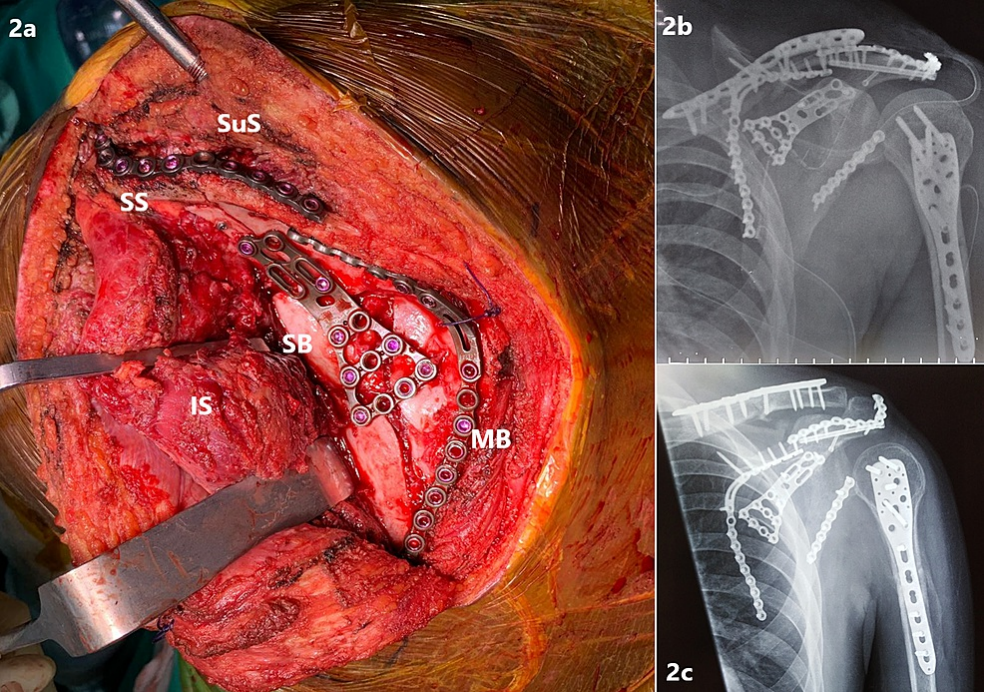
Intraoperative clinical image showing the scapula (a) and the postoperative anteroposterior (AP) radiograph taken at day 2 (b) and six months (c) after the surgery. (a) Intraoperative clinical image showing the posterior surface of the scapula, with the infraspinatus (IS) reflected for adequate exposure to fix the scapular body (SB), spine (SS) and medial border (MB). The supraspinatus (SuS) was also reflected, but not detached completely. The lateral border was accessed using another intermuscular window between the infraspinatus and the teres minor (not shown in the image). (b) The immediate postoperative shoulder AP radiograph shows good reduction and stable fixation of the clavicle, scapula and proximal humerus fracture. (c) A similar view was taken at the follow-up six months after the surgery showing that radiological union of all fractures was achieved, and the fixation remained stable. The glenohumeral joint was slightly luxated, owing to the non-recovery of the brachial plexus injury, causing deltoid and rotator cuff muscle wasting.

After closure and dressing of the anterior wound, the scapula was operated next. A modified Judet approach was adopted. The intermuscular planes between the deltoid and the infraspinatus and between the infraspinatus and the teres minor were developed for adequate exposure to address the complex fractures of the scapula. After initial reduction, the whole scapula was fixed in the following sequence: 1) scapular spine, 2) lateral border, 3) medial border and 4) scapular body. The scapular spine was fixed using a 4.0 mm half-threaded cannulated screw, supplemented by a 2.7 mm anatomical locking plate. Both the borders were fixed using the same sized anatomical locking plate, and the scapular body was fixed using an anatomical buttress locking plate (Figure 2).

There was no intraoperative or immediate postoperative complication. Due to the presence of complete brachial plexus injury, the patient was kept in a sling, and the only physiotherapy regime prescribed for the first six weeks after the surgery was the gravity-assisted pendular exercise. At the sixth week post-surgery, passive range of motion and strengthening exercises were allowed. The fixations were stable throughout the follow-ups; radiological union of all three bones was seen at the third month postoperatively. Despite being pain-free, there was no sign of recovery for his brachial plexus injury, except for a relatively stable shoulder and recovery of some of the trapezius function. After 14 months, the patient is currently under the follow-up of the Hand & Microsurgery Team, with an initial plan for free muscle transfer for elbow flexion being put on hold due to the COVID-19 pandemic and the patient’s indecision on the surgery.

## Discussion

With scapular fracture alone accounting for only 1% of all fractures [1], simultaneous ipsilateral scapula, clavicle and humerus fractures are postulated to be very rare. Although also uncommon, the combination of clavicle and scapula fractures that disrupt the SSSC (floating shoulder), however, is much more well documented [1-5]. Proximal humerus fracture associated with either clavicle or scapula fracture is also rare, as highlighted by Berkes et al., who found only three out of 91 cases of proximal humerus fractures through a prospective database [6]. Hence, the rarity and complexity of simultaneous fractures involving all these three bones, which form the only articulations between the upper limb and the trunk, pose a great challenge in its management.

High-energy trauma, including high-velocity motor vehicle accident and fall from height, is the main established aetiology for fractures about the shoulder girdle, as high as 88% of the time [5]. Associated injuries are not uncommon. In a series of 46 patients sustaining a floating shoulder injury in a multicentre study, only one patient was found to have no other associated injuries [5]. Polytraumas, including head injuries, intra-abdominal haemorrhage and cardiothoracic injuries, are all reported, together with concomitant fractures of the pelvic, spine and lower limb. Adjacent neurovascular injuries are among the dreaded association; as happened in our patient, neural injury such as brachial plexus injury inevitably influences the final outcome. Vascular injuries, especially involving the axillary and subclavian artery, may occur in such trauma and warrant even more urgent attention.

A holistic approach towards patients who present with such complex fractures must be undertaken by the attending doctors. In these trauma patients, initial management must be in line with the Advanced Trauma Life Support (ATLS) protocol: stabilising the airway, resuscitating haemodynamically and identifying and prioritising life-threatening injuries. In a stable patient, focused examination of the injured shoulder and upper limb must always include a detailed neurovascular assessment. Temporary sling or splintage helps alleviate pain and prevents further unwanted damage to soft tissue and neurovascular structures.

Although in most instances plain anteroposterior radiograph is sufficient to establish a diagnosis, additional views such as Zanca, Grashey and scapular Y views help in the assessment of the acromioclavicular joint, glenoid, glenopolar angle and angulation of the scapular body. CT scan is also indispensable for a thorough assessment and for preoperative planning. Angiography by CT scan or magnetic resonance imaging (MRI) is warranted in patients with associated vascular injury. In the acute setting, MRI for the assessment of the ligaments and even brachial plexus might not be suitable due to obscurity by the presence of hematoma and inflammatory response.

The consideration between non-operative and operative management differs greatly between patients who present with isolated scapula, clavicle or humerus fracture and patients who present with all three injuries. In isolated fracture, non-operative management might be the choice in patients with non-displaced or minimally displaced fragments. Although previously non-operative treatment was shown to have satisfactory outcomes for dual injury such as floating shoulder [2,7], many recent publications have favour surgical treatment due to its superior result [3,4].

The increase in understanding of shoulder biomechanics has expanded the indications for surgery. An exhaustive list of indications for floating shoulder includes, among others, displacement of more than 5 and 10 mm for clavicle and scapular neck fractures, respectively; glenopolar angle more than 20°; angulation more than 40°; glenoid involvement with an intra-articular step of more than 3 mm; and neurovascular injury [3-5]. The two subset groups of surgical treatments are those with clavicular fixation only and those with combined clavicular and scapular fixation [2,3]. Comparisons between the two showed no statistical difference in the shoulder functional outcome; the restoration of one component of the SSSC is thought to have stabilised it and improved the function of the other component [3].

The complex nature of the injuries sustained by our patient, combined with brachial plexus injury, inevitably inclined us towards the decision for operative management. The presence of displaced proximal humerus fracture accompanied by the floating shoulder culminated us to fix the fractures to stabilise the shoulder for early rehabilitation and for the ease of any future neural or musculotendinous procedure for his brachial plexus injury. In this case, our choice of positioning the patient in a floppy lateral position was to accommodate our surgical approaches, as we needed access to both the anterior and the posterior aspects of the shoulder. The strategy of the surgical decision adopted was to fix the humerus and clavicle first, from the anterior. By stabilising the humerus first, the ease of mobilisation of the arm helped the subsequent reduction and fixation of the clavicle and scapula.

Generally, in patients without neural injury, early mobilisation of the shoulder equates to good functional outcomes. However, due to the complete brachial plexus injury, wasting of rotator cuff and deltoid muscles leading to luxation of the glenohumeral joint in our patient was unavoidable despite meticulous planning of the rehabilitation regime. In such patients, further treatment might be required and need to be tailored according to the preserved functions and the patient’s potential for recovery.

## Conclusions

The presence of simultaneous ipsilateral clavicle, scapula and humerus fracture is highly suggestive of involvement in high-energy trauma. It is therefore of utmost importance that associated injuries including neurovascular compromise and life-threatening conditions should not be missed. Surgical intervention is usually favoured over conservative management in such cases to facilitate early rehabilitation. Despite its rarity, management of this injury should always be individualised to ensure optimal functional outcomes are achieved.

## References

[1] Herscovici D Jr, Fiennes AG, Allgöwer M, Rüedi TP (1992). The floating shoulder: ipsilateral clavicle and scapular neck fractures. J Bone Joint Surg Br.

[2] van Noort A, te Slaa RL, Marti RK, van der Werken C (2001). The floating shoulder. A multicentre study. J Bone Joint Surg Br.

[3] Zhou Q, Li K, Chen B, Zhou YD, Chen H, Wang Z, Liu JL (2016). [Comparative study on curative effects of different methods for the treatment of the "floating shoulder injuries"]. Zhongguo Gu Shang.

[4] Dombrowsky AR, Boudreau S, Quade J, Brabston EW, Ponce BA, Momaya AM (2020). Clinical outcomes following conservative and surgical management of floating shoulder injuries: a systematic review. J Shoulder Elbow Surg.

[5] Scavenius M, Sloth C (1996). Fractures of the scapula. Acta Orthop Belg.

[6] Berkes MB, Little MT, Pardee NC, Schottel PC, Lazaro LE, Lorich DG (2016). Outcomes of proximal humerus fracture open reduction internal fixation with concomitant ipsilateral shoulder girdle injuries: a case control study. HSS J.

[7] Ramos L, Mencía R, Alonso A, Ferrández L (1997). Conservative treatment of ipsilateral fractures of the scapula and clavicle. J Trauma.

